# Healing through art: a thematic synthesis within a quasi-systematic review of art’s impact on adult mental well-being during the COVID-19 pandemic

**DOI:** 10.1186/s12889-025-22741-0

**Published:** 2025-05-03

**Authors:** Hayley Stevenson, Mamdooh Alzyood

**Affiliations:** 1 Health and Social Care, Warwickshire College Group Leamington Spa College, Warwick New Rd, Leamington Spa, CV32 5JE UK; 2 Public Health, Infection Prevention and Control Consultant, Oxford Brookes University, Headington Campus, Oxford, OX3 0BP UK

**Keywords:** Art therapy, COVID-19, Social isolation, Mental health, Marginalised populations, Digital communication, Resilience, Cultural adaptation, Emotional regulation, Public health, Systematic review

## Abstract

**Aim:**

To synthesise evidence on how both structured art therapy and informal creative engagement supported adult mental well-being during COVID-19-related isolation, and to evaluate their applicability across diverse populations and contexts.

**Method:**

A quasi-systematic review of qualitative studies from CINAHL, PsycInfo, and PubMed (2020–2024), analysed through thematic synthesis.

**Results:**

Analysis of seven studies identified five key themes: (1) emotional processing and expression through symbolic creation, (2) adaptive communication and nonverbal connection, (3) communal support and collective meaning-making, (4) empowerment and regaining agency, and (5) transformation of trauma into post-traumatic growth. Marginalised groups—including isolated elderly individuals, disabled adults, and low-income families—benefited significantly from both formal art therapy and informal artistic activities, which addressed barriers such as limited mobility, communication challenges, and social disconnection.

**Discussion:**

Art-based interventions demonstrate potential as scalable, low-resource tools for mental health support, particularly for individuals experiencing isolation or other forms of vulnerability, such as disability or displacement. However, equitable implementation requires hybrid delivery models and cultural adaptation. While qualitative findings highlight art’s capacity to externalise distress and sustain connection, methodological constraints (small homogenous samples) necessitate mixed-methods validation of long-term outcomes.

**Conclusion:**

Integrating art into public health frameworks could mitigate isolation-related psychological harm, particularly for marginalised groups. Future research should prioritise cross-cultural adaptation of interventions, community-led co-design, and studies that examine how social factors like disability, income, and cultural context intersect to shape the effectiveness of art-based mental health support.

**Clinical trial number:**

Not applicable.

**Supplementary Information:**

The online version contains supplementary material available at 10.1186/s12889-025-22741-0.

## Background

The COVID-19 pandemic is one of the biggest global health crises of our generation [1]. The repercussions for health systems, society and economies have been both severe and long-lasting [2]. In particular, the pandemic has exacerbated pre-existing health and socioeconomic inequalities in education and skills and created new issues for young adults, such as worsening mental health issues [2]. Prolonged quarantine has been linked with deteriorating psychological states [3]. Throughout the quarantine period, individuals voiced apprehensions regarding personal health as well as the potential transmission of infections to others [4]. Additionally, the disturbance of routines and a reduction in social and physical interactions contributed to feelings of boredom, frustration, and disorientation among individuals subjected to lockdown restrictions [3].

Art for well-being is widely accepted; in various forms, art provides meaning and social connection to people’s lives [5]. Research during the COVID-19 pandemic underscored the critical role of creative outlets in mitigating psychological distress, with studies highlighting increased reliance on art-based coping mechanisms during periods of social isolation [6]. For instance, visual arts, music, and writing were increasingly incorporated into telehealth and community mental health interventions, reflecting their adaptability to remote formats [7]. This shift to digital platforms—such as virtual galleries, online art classes, and remote creative workshops—not only expanded accessibility but also fostered global participation in art initiatives [8]. However, while digital engagement offered alternatives to in-person interaction, prolonged isolation exacerbated mental health challenges [9]. Lockdowns disproportionately intensified issues like intimate partner violence (IPV) and gender-based violence (GBV), compounding trauma for vulnerable groups [10–12]. Simultaneously, the dissolution of community-based art groups and in-person classes eroded social support systems, contributing to heightened health anxiety, depression, and stress [13].

In the UK, there has been increased interest in research, practice, academic study, and policies related to art and health. A recent report by the UK All-Party Parliamentary Group on Arts, Health and Wellbeing shows how much taking part in art activities, from professional art treatments to more casual art programmes, can improve personal and public health and well-being [14]. Many reports and reviews emphasise how involvement in art can improve mental health. A systematic review revealed that engaging with art helped people overcome mental challenges and observe life changes that they had previously considered unobtainable [15]. However, it is important to note that despite growing advocacy for the arts in public health, the existing evidence base remains limited and often inconclusive regarding the direct relationship between arts engagement and improved mental well-being [16]. Therefore, this review—through the inclusion of qualitative studies—seeks to offer insight into individuals’ lived experiences and inform the development of a Theory of Change to support the design of future interventions. Although increasing evidence suggests the effectiveness of arts in preventing illness, promoting health, and managing certain illnesses throughout life, it is essential to consider how art can be used as an outlet for emotion and self-expression, and how culturally inclusive or community-based creative initiatives could be introduced to improve the mental well-being of isolated patients within the public health policies [17].

The COVID-19 pandemic has brought unprecedented challenges to mental well-being worldwide, with lockdowns and social restrictions leaving many individuals isolated and struggling to maintain their emotional health [18]. These experiences of isolation and the resulting impact on mental health highlight the importance of creative outlets, such as art, for processing emotions and fostering resilience during difficult times [5, 13]. In this review, ‘mental well-being’ refers to a state of mental health that encompasses emotional, psychological, and social well-being [19]. It involves the ability to manage stress, experience positive emotions, and maintain fulfilling relationships [20]. Specifically, during the COVID-19 pandemic, mental well-being includes the ability to cope with the challenges of isolation [3], articulate complex emotions [21], maintain a sense of connection to oneself and others, and adapt to changes in social environments [22]. Our review examines how art-based interventions may enhance these aspects of mental well-being by providing outlets for emotional expression, fostering social connections, and promoting resilience in the face of uncertainty and stress.

Marginalised groups, operationalised in this review as groups experiencing systemic exclusion due to socioeconomic disadvantage, disability, age, or structural inequities [23], are disproportionately affected during crises such as the COVID-19 pandemic. These groups include elderly individuals in care settings, disabled adults, and low-income households, who often face compounded barriers to accessing mental health resources [24]. Systemic inequities exacerbate their isolation and psychological distress, necessitating interventions that address both structural and individual needs [25, 26].

The cover art accompanying this review was created during the pandemic with my two-year-old son [see Additional file [3]. It represents the chaos and transformation experienced during this period. The mushrooms symbolise the world coming to a halt, allowing life to emerge in unexpected ways, while their life cycle reflects the themes of death, renewal, and growth. The butterflies, trapped within a frame, convey the longing for freedom and the hope for a brighter future, whereas the beach, obscured by a brick wall, symbolises the isolation felt by families confined to their homes. This personal reflection mirrors the experiences captured in the literature reviewed, offering a visual representation of the themes of isolation, adaptation, and the pursuit of well-being during challenging times.

This paper aims to review the literature on the use of art for mental well-being among adults during the COVID-19 pandemic globally. The paper concludes with recommendations for applying these findings in public health policy, education and research.

## Design and methods

### Aim

To review a range of international qualitative studies that have examined the use of art for mental well-being among adults during the COVID-19 pandemic.

### Design

This study adopted a quasi-systematic literature review approach with a thematic synthesis of qualitative data, as described by Thomas and Harden (2008) [27]. The review aimed to synthesise evidence on the role of art engagement—both formal and informal—on adult mental well-being during the COVID-19 pandemic. Although not a full systematic review, this approach involved a structured and transparent search process, predefined eligibility criteria, and a methodical analysis of the findings. The review followed the PRISMA 2020 reporting guidelines [28] to ensure rigour and transparency.

### Eligibility criteria

For the purposes of this review, “art” was defined as the expression or application of human creative skill and imagination, typically in a visual or performative form, such as painting, sculpting, music, dance, and other creative media [29]. To be eligible, studies needed to involve art that was created or engaged with during the COVID-19 pandemic for the purpose of improving mental well-being.

The research question posed in this quasi-systematic review aims to collate insights, experiences, emotions, and opinions from studies completed with people who experienced distress or displacement during the COVID-19 pandemic and who have used the arts specifically to cope; therefore, only qualitative studies have been selected for this literature review.

While the focus of this review was on qualitative evidence, mixed-methods studies were also considered for inclusion if they contained a substantial qualitative component relevant to the research question. In these cases, only the qualitative findings were extracted and synthesised as part of the thematic analysis.

The population, exposure and outcome (PEO) framework was used for the present search and helped in systematically organising the search strategy and inclusion criteria (Table 1).

**Table 1:** The PEO framework

Population (*P*)	Adults aged 18 and over, internationally, during the COVID-19 pandemic.
Exposure (E)	Use of art (painting, music, dance, or any creative activities) during the COVID-19 pandemic.
Outcome (O)	Improvements or changes in mental well-being, including aspects like stress reduction, anxiety relief, or overall mood enhancement.

The topic selected was the impact of the COVID-19 pandemic on the use of art to improve mental well-being in adults. The question was then focused on adults internationally, including those aged 18 years, during the COVID-19 lockdown. The research question has been applied to the PEO framework to ensure that the papers selected will be relevant.

### Search strategy

The databases searched were CINAHL, PsycInfo and PubMed. Relevant MeSH headings and subject headings for the elements of PEO were applied. MeSH headings were created to include Boolean operators, truncation, and wildcards, and the limits applied were title and abstract. To ensure that relevant papers would be available on the topic of art for mental well-being during the COVID-19 pandemic, an initial search was performed on CINAHL. Once the availability of relevant papers was established, a systematic literature review was selected to answer the research question. Two further searches were then conducted on PubMed and PsycINFO.

In addition to the formal database search, supplementary search strategies were used to enhance the robustness of the review. These included reference list screening of included studies and relevant reviews, as well as manual searches of grey literature sources such as institutional reports. While these were not conducted systematically, they helped ensure that relevant studies were not missed.

A combination of keywords and subject headings were used to reflect the PEO structure, including terms such as ‘art’, ‘creative activity’, ‘drawing’, ‘painting’, ‘mental well-being’, ‘emotional health’, ‘adult’, and ‘COVID-19’. A complete list of search terms and Boolean operators used for each database is provided in Appendix A.

### Screening and selection process

The study applied specific inclusion and exclusion criteria to ensure relevance and rigour, as detailed in Table 2. Studies were included if they were in English, focused on primary research, utilised active art methods (drawing or painting, music), and were conducted during the COVID-19 pandemic. Database searches were conducted in January 2025, capturing studies published from January 2020 through to December 2024. Studies that were non-English, secondary research, involved passive art methods, or were conducted before 2020 were excluded.

**Table 2:** Inclusion and exclusion criteria

Inclusion	Exclusion	Rationale
English Language		Author does not speak other languages; translation may introduce bias.
Primary Research		Systematic literature reviews are completed on primary research only.
Art Method - Mark making (drawing, painting, colouring) sculpture, music, dance, sewing/knitting/crochet.	Passive use of art, such as watching theatre on television, virtual exhibitions.	Limitations are applied as interpretation of art engagement is too broad. The act of creating art is researched and evidenced as improving self-esteem, therefore passive engagement was excluded.
Time Frame - During the COVID-19 pandemic (global). Acceptable dates for conducted and published studies 2020–2024.	Pre- 2020.	Any research pre 2020 will not have included the impact that COVID-19 had on mental well-being.

**Fig. 1:**
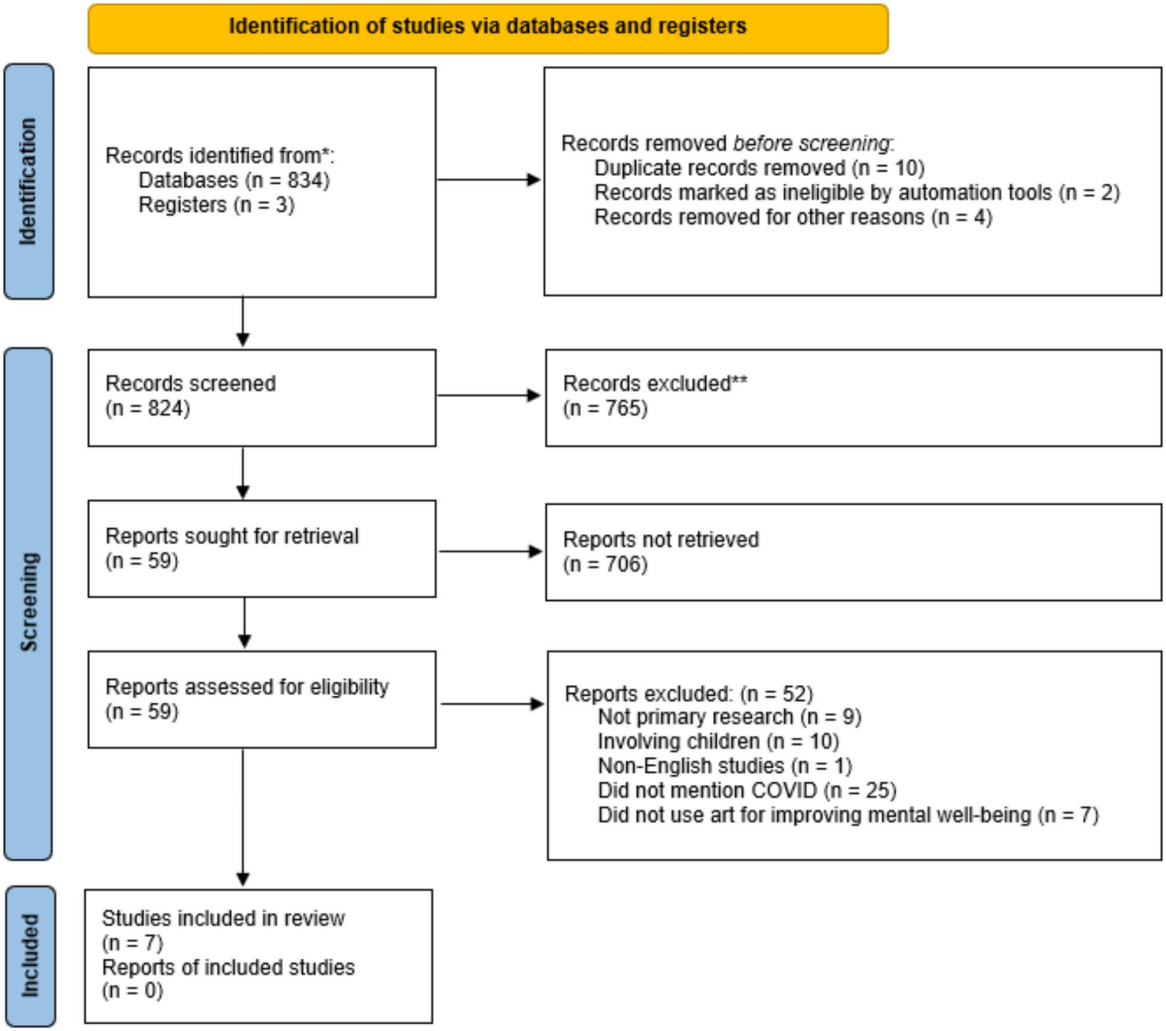
PRISMA flowchart

The PRISMA flow diagram [30] provides a visual representation of the flow of information through this review process, from the initial identification of studies to final inclusion in the review (see Fig. 1).

Screening was conducted in two stages. In the first stage, the titles and abstracts of all retrieved articles were independently screened against the inclusion criteria by the lead author (HS). In the second stage, full texts were assessed for eligibility. To reduce the potential for selection bias, a second author (MA) independently reviewed a random sample (20%) of the full-text articles. Discrepancies were discussed and resolved by consensus. Pre-defined inclusion and exclusion criteria based on the PEO framework were used throughout to maintain consistency and transparency.

### Data analysis

In this review, thematic synthesis, as described by Thomas and Harden (2008) [27], was employed to analyse the qualitative data extracted from the included studies. This approach is well suited for synthesising findings across multiple qualitative studies, allowing for the identification of key themes and patterns. It involves a three-step process that integrates diverse study findings into a coherent synthesis. The first step of the thematic synthesis involved line-by-line coding of the qualitative data, where we carefully coded relevant concepts or ideas from each study. These codes served as labels or categories that summarised significant information from the data. Next, we grouped similar codes to develop descriptive themes, which helped to identify recurring patterns and concepts across the studies. The final step involved refining these descriptive themes into analytical themes, offering new insights and deeper interpretations of the role of art in enhancing mental well-being during the COVID-19 pandemic.

All studies included in this review were imported into NVivo© 14 software, where the highlight and table functions were utilised to organise and summarise codes systematically [31]. This method enabled the clear identification of descriptive and analytical themes, facilitating a deeper understanding of participants’ behaviours, attitudes, beliefs, and experiences. The thematic synthesis approach allowed for a thorough integration of findings, ultimately providing a richer interpretation of the impact of art-based interventions on mental well-being during periods of social isolation.

### Reflexivity

The lead author (HS) is a lecturer in Health and Social Care with professional and academic interests in mental health and therapeutic practices, including the use of creative approaches. During the COVID-19 pandemic, the author engaged in art activities at home, which informed a personal interest in understanding how art may support emotional well-being. This personal experience contributed to the choice of research topic and the sensitivity with which participant experiences were interpreted.

The second author (MA) is a Senior Lecturer and researcher in Public Health, with extensive experience in conducting and supervising qualitative and mixed-methods reviews. Both authors brought different perspectives to the interpretation of the data, contributing to a balanced analysis. To enhance transparency and reduce interpretive bias, the thematic synthesis followed a structured framework and coding decisions were discussed collaboratively.

### Findings

A total of 59 papers were screened, of which 52 were excluded for not meeting the inclusion criteria (see Fig. 1). The key features and main characteristics of the final papers included in this review are summarised and organised in Table 3.

**Table 3:** Main studies’ characteristics

Author(s), year of publication and country of origin	Type of study, sample size, design, analytical method, and inclusion criteria	Purpose of study	Results (only those relevant to review)
Armstrong and Ross, (2021) [33] **UK**,** Dundee**	Mixed methods *N* = 10 Vulnerable parents/infants. Low-income families, parents struggling with isolation. *Methods* ● Thematic analysis of semi-structured interviews and qualitative feedback ● Feedback cards using a quantitative scale and open questions ● Visual data shared (the art images created) ● Semistructured interviews to gather qualitative experience.	To examine the impact of distributing art boxes to support creative interactions between vulnerable parents and their infants during COVID-19 lockdowns.	1. Thematic analysis of interviews revealed that the art boxes supported positive interactions and connections between parents and infants, improving parents’ confidence and emotional well-being. 2. Parents reported increased engagement, playful interactions, and moments of connection, such as through eye contact and shared activities. 3. The art boxes also helped reduce feelings of isolation during the lockdown and enabled parents to continue creative activities independently.
Bungay et al. (2023) [34] **UK**	Qualitative descriptive *N* = 14 *Methods* ● Thematic analysis of open survey responses and haiku poems. ● An online survey was conducted that included both closed- and open-response questions. The questionnaire also invited respondents to write a haiku poem on their experiences, which was used in the analysis. ● Individual interviews	To explore how and why staff and students in one UK university engaged with arts and creative activities during the UK lockdowns and how it impacted their mental well-being.	1. Participants improved their mental wellbeing through a sense of achievement. 2. Connecting with others 3. The activities were used to deal with loss and uncertainty caused by the virus. 4. The value of engagement in the arts during restrictions 5. Discussed social restrictions/isolation and future implications for policy makers
Elisondo and Fernanda Melgar (2020) [35] **Argentina**	Qualitative exploratory *N* = 25 (20 women and 5 men) aged between 22 and 49 years. *Method* ● Grounded theory with open coding using ATLAS.ti software; data triangulation performed. ● Online questionnaire with open-ended questions	To explore how people engaged in creative activities during the COVID-19 pandemic and how these activities transformed their everyday lives, work, and leisure during periods of total and partial confinement.	1. Participants engaged in various creative activities during quarantine, which helped generate positive emotions and cope with negative ones. 2. Creative activities were reported to foster resilience and adaptability, allowing participants to find new ways to manage challenges and transform their routines during the pandemic.
Geréb et al. (2021) [36] **Hungary**	Qualitative exploratory *N* = 22 International university students, aged 21–35 years, from eight countries studying in Hungary. *Methods* ● Thematic coding by three independent researchers analysing artwork, reflective writing, and task responses. ● A qualified art therapist and clinical psychologist designed a series of 7 art-making tasks guided by evidence-based art therapy techniques for self-help. ● Three researchers with MA-level psychology degrees coded the images by theme and symbols. The first and last authors led the data collection and coauthors reviewed the findings and agreed on themes. ● Atlas.ti software used to assign codes based on the image and reflections.	To explore how online self-help art therapy-based tasks could support international students during the COVID-19 lockdown, focusing on their emotional responses, coping strategies, and experiences of isolation and support.	1. Three primary themes emerged: (1) feelings of isolation, (2) loss of control, and (3) seeking support through art-making. 2. Art therapy-based tasks provided a means for participants to manage stress and express their emotions, helping them cope with the challenges of lockdown. 3. The online format allowed students to reflect on their feelings and experiences, offering insights into their need for attachment, connection with nature, and finding inner strength during the pandemic.
Renzi et al. (2020) [37] **Italy**	Qualitative case-study *N* = 1 Elderly woman (age 77) living in a nursing home in Rome, Italy. 77-year-old woman, isolated due to lockdown. *Methods* ● Descriptive narrative and interpretive commentary based on participant artwork and thematic progression. ● A psychologist monitored the participants mental state through weekly counselling sessions and by interpreting themes of the artwork the participant was asked to create as a means of coping with isolation. A report was then published.	To explore how drawing could be used as a therapeutic intervention for expressing and processing emotions during the COVID-19 pandemic for older adults in isolation. The goal was to understand how art could help mitigate feelings of loneliness and support emotional expression when face-to-face social interactions were not possible.	1. The use of drawing helped the participant to explore and represent her emotional state during isolation. 2. Her drawings evolved over time, reflecting her journey from feelings of deep loneliness and separation to a more positive emotional state as restrictions eased. 3. The activity provided a medium for the participant to express her hopes, fears, and feelings of confinement, offering therapeutic benefits that supported her overall emotional well-being.
Said Houari and Hadjoui (2022) [38] **Algeria**	Qualitative case-study *N* = 1 Disabled individual. Adult with West Syndrome, confined at home. *Methods* ● Thematic interpretation based on Malchiodi’s and Kramer’s art therapy theories; inferred outcomes from observation and caregiver interviews. ● A single participant with West Syndrome. ● The data was analysed using Malchiodi’s theory of ‘art *in* therapy’ and Kramer’s theory of ‘art *as* therapy’ ● Five-week art therapy intervention conducted at home. ● Observation, art therapy sessions, and pre- and post-intervention interviews.	To assess how art therapy could be used as a coping strategy for individuals with disabilities during confinement. It focused on exploring the effects of art therapy on emotional well-being and resilience, particularly for those isolated due to COVID-19 restrictions.	1. Art therapy helped the participant express emotions and reduced stress levels during confinement. 2. The sessions improved the participant’s mood, facilitated communication through storytelling and role-play, and supported family connections. 3. Art activities like drawing and clay work allowed the participant to externalise emotions and engage in creative self-expression, which contributed to a more positive emotional state during the period of isolation.
Usiskin and Lloyd (2020) [39] **UK/France**	Qualitative case-study N = NA *Methods* ● Thematic narrative analysis of collaborative artwork and participant reflection in virtual quilting projects (no structured framework reported). ● The use of grounding techniques developed from the principles of Psychological First Aid. ● Online work with participants using trauma-informed approaches. ● Using the window of tolerance with participants and in-depth reporting.	To adapt art therapy to support social engagement and well-being across borders during the COVID-19 pandemic, using online platforms. It sought to create safe spaces for displaced individuals through creative practices, helping them process trauma and build resilience despite physical isolation.	1. Online art therapy sessions provided a sense of connection and reduced feelings of isolation among participants. 2. Highlighted the benefits of art therapy in offering emotional support, fostering community, and helping participants cope with the uncertainty and challenges of the pandemic through creative expression.

The seven studies included in this review were conducted across a range of international settings, including the United Kingdom, France, Argentina, Algeria, and Hungary. Most studies adopted qualitative methodologies, with two designed as case studies and one using a mixed-methods approach. Sample sizes varied considerably, ranging from single case reports to qualitative studies involving over 100 participants. The studies explored a range of art forms, including drawing, sculpture, music, poetry, and mixed media, with some interventions delivered in-person and others virtually. Participants included parents, older adults, disabled individuals, university students, and refugees, all of whom experienced varying degrees of social isolation during the COVID-19 pandemic. Thematic or narrative analysis was commonly employed to examine participants’ experiences and emotional responses to the creative process.

### Thematic overview

The analysis revealed five interconnected themes that elucidate how art-based interventions mediated mental well-being during the COVID-19 pandemic. For clarity, each theme is introduced with a short explanation of the specific terminology and conceptual focus. Synthesised from participants’ lived experiences, these themes reflect the multifaceted mechanisms through which art helped to alleviate isolation, articulate unspoken distress, and nurture psychological adaptability. These themes are: (1) emotional processing and expression through symbolic creation, which explores how participants externalised complex emotions into tangible forms; (2) adaptive communication and nonverbal connection, highlighting art’s role in bridging communication gaps for marginalised groups; (3) communal support and collective meaning-making, emphasising collaborative practices that fostered solidarity; (4) empowerment and regaining agency, detailing how structured creative tasks restored participants’ sense of control; and (5) transformation of trauma into post-traumatic growth, illustrating how adversity was reframed into resilience. The following sections explore these themes in depth, supported by findings from the included studies.

To enhance the transparency and rigour of the thematic synthesis, Table 4 presents selected quotes from the included studies alongside the initial codes and final themes to which they contributed. This table illustrates how the synthesis process was grounded in the original data and how interpretive insights were developed. These examples demonstrate how the voices of participants were central in shaping the key findings of this review. For a comprehensive account of supporting quotes, codes, and themes, please refer to Appendix B.

**Table 4:** Summary of themes with example codes and quotes

Theme	Illustrative code	Illustrative quote	Source/reference
1. Emotional Processing and Expression through Symbolic Creation	Turning distress into manageable visual form	“*The task gave me an opportunity to turn my frustrations into something more manageable*.”	[36]
	Letting go through symbolic art	“*I allowed myself to accept the memory and to let go a little*.”	[36]
2. Adaptive Communication and Nonverbal Connection	Nonverbal understanding	“*Art helped us understand each other without speaking… it was just there*,* visible*.”	[33]
	Digital image-sharing as emotional expression	“*We shared images on social media and it felt like we were speaking the same language*.”	[33]
3. Communal Support and Collective Meaning-Making	Shared art as mutual support	“*We created something together that we could show others. That gave me strength*.”	[34]
	Collective recognition and meaning	“*Even online*,* I felt part of something– it was more than just drawing*,* it was being seen*.”	[37]
4. Empowerment and Regaining Agency	Feeling empowered through creation	“*It provides me with feelings of control*,* power*,* and self-confidence*.”	[36]
	Regaining purpose through art	“*Doing something with my hands helped me feel useful again*.”	[38]
	Emotional transformation through drawing	“*My feelings became less scary. The process of drawing relaxed me*.”	[36]
	Visual marker of progress and growth	“*I look at my painting and see how far I’ve come. It’s proof of healing*.”	[35]

#### Emotional processing and expression through symbolic creation

This theme examines how participants used art as a means to transform abstract and complex emotions into tangible symbols. In this context, ‘*symbolic creation’* refers to the process of externalising internal feelings into visual or tactile forms, which aids in processing distress and reconnecting with one’s sense of self.

Participants used symbolic creation to convert abstract psychological states—such as loneliness, grief, and anxiety—into visual or tactile forms. For example, an elderly woman in a nursing home depicted her longing for freedom during lockdown through a drawing titled *Beyond the Hedge*, which symbolised her confinement behind institutional barriers and her hope for liberation [32]. Similarly, international students engaged in guided art tasks externalised feelings of disconnection through metaphors like ‘*storms’* and ‘*extinguished fires’* in their artwork, translating pandemic-induced isolation into a shared visual language [33]. For instance, a student reported “*The task gave me an opportunity to turn my frustrations into something more manageable. My feelings became less scary. The process of drawing relaxed me*” [33]. These symbolic acts provided participants from the included studies with a structured means to confront emotions that words alone could not capture.

Creative practices also helped participants express and process their emotional experiences during confinement. In one case study, an elderly woman residing in a nursing home used drawing to explore her sense of loneliness and isolation during the pandemic [32]. Through her artwork, she depicted a landscape with a closed window, which her psychologist interpreted as symbolising the desire for connection with her social group. The act of drawing supported emotional exploration and helped alleviate depressive symptoms, offering a therapeutic outlet during an otherwise isolating experience [32]. Similarly, a disabled adult with West Syndrome used clay modeling to express emotions nonverbally, reclaiming agency over her narrative despite communication barriers exacerbated by confinement [34]. These cases illustrate how art became a tool for self-reaffirmation, enabling participants to preserve continuity between their past and present selves.

Art-based activities also served as a coping mechanism, providing an outlet for emotional exchange and sustaining social ties, as shown in two studies [35, 36]. Participants engaged in artistic endeavours to communicate feelings when face-to-face interactions were limited. For instance, individuals in Argentina shared digital works to stay linked with friends and family during lockdowns, reducing disconnection [36]. University staff and students in the UK noted that cultural activities like crafting and poetry-writing fostered continuity and shared purpose, countering lockdown loneliness [35]. These activities, ranging from crafting to writing poetry, were particularly effective in helping participants process their emotions and foster a sense of social connection despite the lack of face-to-face interactions.

#### Adaptive communication and nonverbal connection

This theme highlights how art provided alternative communication channels, especially for marginalised groups or those with inherent communication barriers. ‘*Adaptive communication*’ in this context involves utilising non-verbal forms of dialogue where creative outputs complement or substitute spoken language.

For populations with communication barriers, art became a critical tool for dialogue. Parents in low-income households engaged in art box activities with their infants, interpreting nonverbal cues like eye contact and gestures during collaborative painting sessions. These interactions deepened emotional attunement, counteracting the stress of pandemic parenting [37]. Similarly, a disabled adult with West Syndrome, confined to her home, employed clay modeling and music to articulate emotions that words could not convey. Her caregivers reported improved responsiveness to her needs, as her creative output provided tangible insights into her emotional state [34]. Art became a powerful alternative to verbal expression for some individuals: “*The art works realised during home confinement enabled Farah to get in touch with feelings that she could not easily express in words. Painting thus acted as a ‘container’ for negative emotions.*” [34]. These cases highlight art’s role in bridging communication gaps exacerbated by physical and social constraints.

Virtual platforms expanded access to creative communities for geographically isolated groups. International students participating in online art therapy depicted isolation in digital collages, fostering peer solidarity despite physical separation [33]. Meanwhile, displaced individuals in Art Refuge’s virtual sessions shared culturally significant objects (family heirlooms) through screen-based storytelling, transforming digital spaces into transnational hubs of symbolic connection. Art-making was described as a vital source of emotional connection and cultural continuity, especially for displaced individuals who used the virtual art table to maintain links with their heritage and sense of belonging across borders [38].

#### Communal support and collective meaning-making

This theme focuses on the power of collaborative creative practices to generate shared meaning and foster community resilience. ‘*Collective meaning-making*’ refers to the process of integrating individual experiences into a broader narrative of endurance, thereby reinforcing communal bonds.

Families used art to reframe pandemic stressors into opportunities for bonding. Parents involved in the art box project described the collaborative painting sessions with their infants as uplifting moments that broke the monotony of lockdown [37]. These sessions often evolved into intergenerational activities as siblings and other family members joined, creating shared creative rituals that supported familial connection and well-being [37]. In Argentina, adults confined to small living spaces engaged in shared creative activities—such as crafting, poetry, and storytelling—to maintain emotional connection and familial cohesion during lockdown [36]. These practices were described as meaningful strategies to cope with the emotional burden of confinement and to foster a sense of unity within households [36].

Global art initiatives fostered solidarity beyond physical borders. The Coronaquilt project, a collaborative digital artwork, enabled participants across borders to visually share their pandemic experiences, fostering a sense of communal resilience [38]. University students and staff engaged in haiku poetry as a way to process collective grief and connect emotionally during isolation. The shared virtual platform for these poems fostered a communal sense of support and solidarity [35].

#### Empowerment and regaining agency

This theme explores how structured creative tasks enabled participants to reclaim a sense of control over chaotic environments. Here, ‘*empowerment*’ refers to the restoration of personal agency—both spatially and emotionally—by using art to create order and spaces for self-expression.

Repetitive creative acts imposed order on overwhelming routines. University staff described daily knitting sessions as “meditative,” offering a calming rhythm that helped them manage the stress of remote work during the pandemic [35]. In a nursing home, an elderly participant engaged in drawing to express her emotions during isolation. The artwork became a channel for hope and self-reflection, according to the attending psychologist [32].

Art enabled participants to create both mental and physical sanctuaries during periods of confinement. In Argentina, adults living in small spaces used art to symbolise emotional escape, with some depicting beaches obscured by images of brick walls to represent pandemic restrictions [36]. In another study, a disabled participant engaged in music-movement therapy began to demonstrate greater self-confidence and agency. Her family noted that she transitioned from passive participation to taking an active role in directing the sessions, which they described as a marked shift in her self-expression [34].

#### Transformation of trauma into post-traumatic growth

This final theme examines how art facilitated the reconfiguration of traumatic experiences into opportunities for growth. ‘*Transformation of trauma*’ involves using creative expression as a means to reinterpret adversity, thereby fostering resilience and self-efficacy.

Participants often transformed their emotional distress into hope through metaphorical and symbolic expression. In virtual art therapy sessions, refugees used imagery such as phoenixes rising from ashes to reframe their experiences of displacement as narratives of survival and renewal [38]. Similarly, university students facing lockdown anxiety composed haiku poetry, finding the structured form helpful in processing overwhelming emotions [35]. The act of creating provided participants with a sense of narrative control, allowing them to reframe fear and uncertainty into something more tangible and expressive. In one study, a participant described the process as an opportunity to turn frustration into something more manageable [33].

Creative skill development appeared to support long-term confidence and psychological growth. In one case, a disabled participant with West Syndrome demonstrated increased communication and emotional expression through clay modelling and music-based art therapy, which was later reflected in her daily interactions and behaviours [34]. Similarly, an elderly woman living in a nursing home used drawing as a means of emotional exploration during isolation. Her artwork progressed from enclosed, confined spaces to more open, hopeful imagery, which her psychologist interpreted as a positive shift in emotional state and outlook [32].

## Discussion

Findings from this quasi-systematic review elucidate the multifaceted role of art-based interventions in mediating mental well-being during the COVID-19 pandemic. Our analysis identified five interconnected processes through which these interventions appeared to support psychological well-being: emotional processing via symbolic creation, adaptive nonverbal communication, communal meaning-making, empowerment through agency restoration, and post-traumatic growth. The themes identified from the review highlighted several aspects of art-based approaches—such as emotional expression, nonverbal communication, and creative autonomy—that offer alternative pathways for supporting well-being. These approaches could benefit from further study to explore how they complement or extend conventional therapeutic support, particularly in contexts of marginalisation or isolation. Several studies included in this review focused on individuals who may be considered vulnerable or marginalised—such as those experiencing disability, isolation, or displacement. Art-based interventions appeared particularly valuable in these contexts, while also showing potential benefits for broader adult populations.

It is important to distinguish between cultural arts and art therapy in this context. Cultural arts encompass a diverse range of artistic practices grounded in cultural traditions [21], whereas art therapy represents a structured intervention guided by psychological principles [39]. Both approaches, however, have proven to be valuable tools for enhancing mental well-being, particularly during periods of social isolation [33, 36]. The following sections discuss each of these key mechanisms in detail, highlighting their contributions to mental well-being and their implications for future interventions.

Participants transformed intangible psychological distress—loneliness, grief, uncertainty—into tangible forms through metaphor and imagery. For instance, in one case study, art-based practice helped an elderly individual rediscover meaning and continuity during isolation using drawings like Beyond the Hedge [32] to externalise confinement, while refugees redefined trauma through ‘*rebirth*’ symbolism [38]. This aligns with theories positing that art externalises internal states, enabling cognitive distancing and mastery over adversity [21, 39]. However, our findings uniquely highlight how pandemic-induced isolation intensified reliance on such symbolic processes, particularly among groups with limited verbal or digital communication access (disabled adults [34], elderly care home residents [32]).

Art circumvented communication barriers exacerbated by lockdowns, fostering connection where words fell short. Parents interpreting infants’ nonverbal cues during art box sessions [37] and displaced groups sharing heirlooms in virtual therapy [38] exemplify art’s role in sustaining relational bonds amid physical separation. These findings extend prior research on digital mental health tools [7] by emphasising art’s unique capacity to convey cultural and emotional nuance in low-resource settings—a critical consideration for groups excluded from text- or language-based interventions.

Collaborative projects like the Coronaquilt [38] and familial co-creation rituals [36, 37] transformed individual struggles into shared narratives, fostering solidarity across geographic and generational divides. This resonates with sociological frameworks that position collective art-making as a form of ‘social prescription’ [14], mitigating isolation through purposeful collaboration. Collective art-making has been theorised as a social health resource that fosters a sense of belonging, connectedness, and purpose [40, 41]. Additionally, participatory arts have been integrated into public health strategies to address loneliness and improve community resilience [42]. Our review underscores the necessity of adaptable communal practices: while digital platforms expanded access, marginalised groups with limited connectivity (low-income families [37]) relied on low-tech, household-based activities, suggesting a need for hybrid intervention models.

Beyond its therapeutic function, collective art-making can also be viewed as a form of cultural resistance and collective agency. During the COVID-19 pandemic, art provided marginalised individuals and groups with a means of reclaiming visibility, asserting identity, and resisting narratives of passivity or isolation [43, 44]. Community-created artworks, including digital exhibitions and collaborative murals, became acts of defiance against social fragmentation, turning creative expression into a vehicle for solidarity, empowerment, and socio-political voice. This aligns with theoretical perspectives that view participatory art as a practice through which individuals and groups enact agency, challenge marginalisation, and co-create meaning during times of crisis [45, 46].

Structured creative tasks helped some individuals restore a sense of agency by ritualising chaos into manageable routines. In one case study, a university staff member described daily knitting as a grounding ritual that reduced stress during lockdown [35]. Likewise, a single elderly participant used regular drawing sessions to process emotions and introduce structure into her isolated days [32]. Although the evidence is limited, these examples echo behavioural theories linking repetitive motion to anxiety reduction [20]. Our findings also suggest that art’s flexible structure—balancing predictability with creative freedom—may cater to different needs. For instance, Argentinian adults in cramped homes used art to carve symbolic ‘escape routes’ [36], while a disabled participant in a music-movement therapy programme developed greater confidence in self-expression and agency [34]. While these accounts are illustrative rather than conclusive, they highlight art’s potential dual role as both a stabilising force and a means of reclaiming autonomy.

Art facilitated post-traumatic growth by reframing adversity into narratives of resilience. Refugees in the Art Refuge project used creative expression to communicate displacement, identity, and hope, contributing artwork that served as symbolic representations of emotional survival and solidarity [38]. Similarly, students’ haiku poetry offered a way to impose structure on uncertainty, aligning with theories of narrative identity reconstruction [35]. Notably, marginalised groups leveraged art not just to cope but to redefine their societal roles—a disabled participant’s transition from passive patient to active self-advocate [34] illustrates art’s potential to challenge stigmatising labels.

### Summary of findings and implications

This review demonstrates that art-based interventions functioned as a multifaceted tool for supporting mental well‐being during the COVID‐19 pandemic. By enabling individuals to externalise complex emotions through symbolic creation, art transformed abstract psychological distress—manifesting as loneliness, grief, and anxiety—into tangible forms, thereby fostering emotional clarity and cognitive mastery. For marginalised groups facing communication barriers, art bridged nonverbal and digital divides by offering alternative channels or processes to express needs and maintain relational bonds amid physical isolation. Furthermore, collaborative creative practices nurtured communal resilience by reframing individual struggles into shared narratives of solidarity, as evidenced by initiatives such as collective quilts and family co‐creation rituals. Structured creative tasks restored agency by ritualising chaos into manageable routines, empowering participants to reclaim autonomy within constrained environments. Crucially, art facilitated post‐traumatic growth by enabling individuals to reinterpret adversity as a catalyst for resilience, skill development, and renewed self‐efficacy.

### Strengths and limitations

A key limitation of this review is its reliance on small-scale qualitative studies, predominantly case reports and exploratory designs. While these offer rich, context-specific insights into individual experiences, their findings are not readily generalisable to wider populations. The heterogeneity in study methodologies, participant demographics, and intervention types further complicates efforts to synthesise results or draw firm conclusions regarding the overall effectiveness of art-based interventions. Although individual case studies provided suggestive evidence of benefits for elderly and disabled individuals, these findings should be interpreted with caution. Further research is required to assess whether such interventions could benefit wider marginalised or general populations.

Another important constraint lies in the nature of the data synthesis. Thematic synthesis was conducted on secondary data—that is, the authors’ interpretations and selected participant quotations from the included studies—rather than full access to raw qualitative data such as transcripts or complete artwork analyses. While this method is consistent with accepted approaches to qualitative synthesis, it does limit the depth and granularity of the thematic analysis, as it relies on what was reported by original study authors.

Despite these limitations, the review offers a systematic and nuanced account of how art-based interventions may support mental well-being, particularly in the context of pandemic-related social isolation. Several included studies highlight how creative engagement can foster emotional connection, support expression, and contribute to psychological resilience. These findings underscore the potential of art-based approaches to promote biopsychosocial well-being and suggest areas for further exploration. Future research should prioritise rigorous, mixed-methods designs with diverse samples to better understand the processes through which artistic expression may alleviate stress and enhance mental health outcomes. Ensuring that such interventions are accessible, culturally sensitive, and inclusive will be key to informing equitable public health strategies.

### Recommendations for policy, education, and research

Policymakers should consider developing well-being guidelines within health and social care systems that embed access to art and creative activities as part of broader mental health strategies. This aligns with international calls for social prescribing and community-based arts interventions to support population mental health [14, 47]. Creative programmes have been shown to reduce loneliness, improve self-esteem, and strengthen community cohesion — particularly when tailored for marginalised groups or individuals experiencing social isolation [48, 49].

Health institutions could benefit from partnering with professional artists and art therapists to integrate art-based interventions into existing mental health services. These initiatives may enhance person-centred care, provide non-pharmacological options for emotional expression, and promote recovery-oriented practices [50]. Investment in staff training around creative facilitation, along with access to appropriate materials and resources, would also be essential for sustainable delivery.

In educational institutions — particularly universities, where loneliness and mental health concerns are increasingly prevalent — the introduction of communal spaces for creative expression could serve as a cost-effective early intervention strategy [51]. Such spaces may offer inclusive, non-clinical environments that reduce stigma and foster peer support through shared artistic engagement.

Future research should further investigate the integration of art within health and community settings, with particular attention to its impact across diverse demographic groups. Mixed-method and longitudinal designs could provide more robust evidence of effectiveness and guide scalable, evidence-informed policy implementation.

## Conclusion

Creative interventions demonstrate significant potential for marginalised groups, including isolated elderly individuals in care settings, disabled adults with communication barriers, and low-income families in constrained environments. These interventions may be particularly valuable for individuals in vulnerable situations, although they also hold promise for wider populations. By alleviating isolation through structured creative tasks, collaborative projects, and nonverbal expression, art therapy addresses challenges exacerbated by physical separation. Although this review focuses on pandemic lockdowns, its implications extend to contexts such as forced migration, chronic illness, and institutional care, where prolonged disconnection further intensifies psychological distress. These findings advocate for the broader integration of art-based strategies into mental health care, alongside rigorous research to optimise their applicability across diverse isolation scenarios and cultural contexts.

## Electronic supplementary material

Below is the link to the electronic supplementary material.

Supplementary Material 1

Supplementary Material 2

Supplementary Material 3

## Data Availability

All data generated or analysed during this study are included in this published article and its supplementary information files.
