# Supplementary material for: Healing through art: a thematic synthesis within a quasi-systematic review of art’s impact on adult mental well-being during the COVID-19 pandemic
Source: BMC Public Health. 2025 May 3;25:1641. doi: 10.1186/s12889-025-22741-0 (PMC12048940; doi:10.1186/s12889-025-22741-0)
Supplement: Supplementary file 1 — Supplementary Material 1 [file 12889_2025_22741_MOESM1_ESM.docx]

**Appendix A**: Database search strategies

| **Database** | **Search String (using MeSH and free-text terms):** | **Limits applied** |
| --- | --- | --- |
| PubMed search strategy | ("Art"[MeSH Terms] OR "Art Therapy"[MeSH Terms] OR "art-based intervention*" OR "creative activit*" OR "visual art*" OR "community art" OR "arts engagement" OR "art practice*" OR "art project*" OR "drawing" OR "painting" OR "sculpture")  AND  ("COVID-19"[MeSH Terms] OR "Coronavirus"[MeSH Terms] OR "SARS-CoV-2" OR "pandemic*" OR "lockdown*" OR "quarantine")  AND  ("Mental Health"[MeSH Terms] OR "mental well-being" OR "emotional well-being" OR "psychological health" OR "stress" OR "anxiety" OR "depression" OR "resilience")  AND  ("Adult"[MeSH Terms] OR adult* OR "young adult*" OR "older adult*") | - Publication dates: 2020 – 2024 - Humans - English language |
| PsycINFO (via EBSCOhost) Search Strategy | (AB("art therapy" OR "art-based intervention*" OR "creative activit*" OR "arts engagement" OR "visual art*" OR "drawing" OR "painting" OR "community art" OR "art project*"))  AND  (AB("COVID-19" OR "pandemic*" OR "lockdown*" OR "quarantine" OR "Coronavirus" OR "SARS-CoV-2"))  AND  (AB("mental health" OR "mental well-being" OR "psychological well-being" OR "emotional regulation" OR "stress" OR "anxiety" OR "depression" OR "resilience"))  AND  (AB("adult*" OR "young adult*" OR "older adult*")) | - Peer-reviewed journals - English language - Publication year: 2020 – 2024 |
| CINAHL (via EBSCOhost) Search Strategy | ("art-based intervention*" OR "art therapy" OR "creative activit*" OR "visual art*" OR "arts in health" OR "drawing" OR "painting" OR "community art" OR "art project*")  AND  ("COVID-19" OR "pandemic*" OR "lockdown*" OR "quarantine" OR "Coronavirus" OR "SARS-CoV-2")  AND  ("mental health" OR "mental well-being" OR "emotional health" OR "psychological health" OR "resilience" OR "stress" OR "anxiety" OR "depression")  AND  ("adult*" OR "young adult*" OR "older adult*") | - Full text - English language - Research articles - Date range: 2020 – 2024 |
