# Supplementary material for: Healing through art: a thematic synthesis within a quasi-systematic review of art’s impact on adult mental well-being during the COVID-19 pandemic
Source: BMC Public Health. 2025 May 3;25:1641. doi: 10.1186/s12889-025-22741-0 (PMC12048940; doi:10.1186/s12889-025-22741-0)
Supplement: Supplementary file 2 — Supplementary Material 2 [file 12889_2025_22741_MOESM2_ESM.docx]

**Appendix B:** Thematic synthesis coding framework – Progression from participant data to final analytical themes

| Study 1: Armstrong & Ross, 2021 | Art boxes supporting parents and infants to share creative interactions at home during COVID-19 lockdown | | |
| --- | --- | --- | --- |
| **Participant Quote or Description (from the study)** | **Initial Code** | **Descriptive Theme** | **Final Analytical Theme** |
| Parents “reported feeling bored themselves alongside guilt about their infant's experiences.” | Parental emotional strain | Emotional burden during isolation | Emotional processing and expression through symbolic creation |
| “All parents reported feeling more confident from using the box… now able to adapt activities to suit or come up with ideas.” | Increased parental confidence | Skill-building and self-efficacy | Transformation of trauma into post-traumatic growth |
| “[The activities] helped them to feel they were doing a good enough job” | Reassurance and validation | Enhancing parental self-worth | Empowerment and regaining agency |
| “All the parents reported feeling connected to their infant during the art activities.” | Parent-infant connection | Shared emotional moments | Communal support and collective meaning-making |
| “Parents described being more playful with their infant during the art.” | Playful interaction | Creative engagement enhancing bond | Communal support and collective meaning-making |
| “Infants were happy, having fun and livelier.” | Child enjoyment and emotional response | Positive child response to art | Emotional processing and expression through symbolic creation |
| “Infants enjoying the consequences of their actions with the materials.” | Developing agency | Sense of autonomy in infants | Empowerment and regaining agency |
| “Several mentioned not having ‘mum friends’… no opportunity to make them.” | Social isolation | Maternal loneliness during lockdown | Adaptive communication and nonverbal connection |
| “All brought up other ways in which they had shared art works… sharing images over social media.” | Digital sharing of artwork | Connecting beyond the household | Adaptive communication and nonverbal connection |
| “One parent made this explicit saying that they felt ‘blessed’ and that they were thought of.” | Feeling seen and supported | Symbolic social recognition through receipt of the box | Communal support and collective meaning-making |
| “Parents talked about having art works up on display and of particular things that they would keep.” | Tangible outcomes of art | Art as memory and emotional anchor | Emotional processing and expression through symbolic creation |
| “They all felt they had needed the instruction booklet… particularly for guidance around what works for younger ages.” | Instruction as empowerment | Gaining practical competence through structured guidance | Empowerment and regaining agency |
| Study 2: Bungay et al. (2023) | Arts and creativity: maintaining mental wellbeing during COVID-19 lockdowns in UK universities | | |
| **Participant Quote or Description (from the study)** | **Initial Code** | **Descriptive Theme** | **Final Analytical Theme** |
| “I found knitting very therapeutic... something rhythmic about it helped me to unwind.” | Rhythmic creative acts reduce stress | Repetitive practice offers relief from anxiety | Empowerment and regaining agency |
| “The simple act of making something gave me a sense of purpose when everything else felt chaotic.” | Creating gives structure and meaning | Craft as a grounding, stabilising force | Empowerment and regaining agency |
| “When I was painting, I could forget the news and just be in the moment.” | Artistic absorption offers escape | Temporary mental refuge through art | Emotional processing and expression through symbolic creation |
| “It gave me a sense of connection – even though we were physically apart, we were doing this together.” | Feeling connected despite physical isolation | Shared creative practices foster belonging | Communal support and collective meaning-making |
| “I didn’t think I was creative, but doing these tasks helped me realise I had a voice.” | Discovering latent creative identity | Realising self-expression through art | Transformation of trauma into post-traumatic growth |
| “Writing a haiku about how I was feeling helped me clarify my thoughts.” | Writing as a tool for emotional clarity | Art enables articulation of complex feelings | Emotional processing and expression through symbolic creation |
| “It helped break the monotony... gave the day some shape.” | Creative activity gives structure | Ritualised art breaks up the day and routine | Empowerment and regaining agency |
| “Seeing others’ haikus made me feel less alone in how I was feeling.” | Creative outputs as shared experience | Collective expression creates solidarity | Communal support and collective meaning-making |
| “We were able to connect without needing to explain everything.” | Connection without words | Art enables nonverbal emotional communication | Adaptive communication and nonverbal connection |
| Study 3: Elisondo (2022) | Everyday Creativity in Times of COVID-19: A Qualitative Study from Argentina. | | |
| **Participant Quote or Description (from the study)** | **Initial Code** | **Descriptive Theme** | **Final Analytical Theme** |
| “The creative things we did were the vegetable garden, the new recipes, the games to entertain ourselves and ways of doing physical activity in our little house.” (Vanesa) | Home-based creative adaptation | Restructuring daily routines through creativity | Emotional processing and expression through symbolic creation |
| “We had to accommodate the spaces and recondition technologies. We also had to create schedules and organize the times...” (Maria) | Managing space and time | Negotiating domestic environments during isolation | Empowerment and regaining agency |
| “We are always looking for alternatives and creative ways to feed more people in the NGO...” (Susana) | Altruistic creativity | Solidarity-based creative practices | Communal support and collective meaning-making |
| “I wrote a lot of new music and put together a publication plan...” (Juan) | Creative entrepreneurship | Reimagining identity through new ventures | Transformation of trauma into post-traumatic growth |
| “Because my mother couldn’t go to work... we started selling surprise breakfasts.” (Maria) | Economic adaptation through creativity | Creative resilience during financial strain | Empowerment and regaining agency |
| “Fear, despair, sadness... uncertainty, fatigue...” (Carla) | Emotional volatility | Emotional flux during confinement | Emotional processing and expression through symbolic creation |
| “I am happy to be at home... have time to learn and do new things...” (Cristina) | Rediscovery of joy in creative acts | Positive emotion through engagement | Post-traumatic growth through creativity |
| “Crossed emotions... the fact of starting a career... puts me in a very good mood.” (Mariano) | Delayed aspirations | Unlocking potential through postponed goals | Transformation of trauma into post-traumatic growth |
| “I contacted the parish... had a strong need to collaborate... helping has helped me to be a little better.” (Emma) | Solidarity and healing | Altruistic creative acts for emotional wellbeing | Communal support and collective meaning-making |
| “Yoga, gardens, meditation spaces... organic products, food for sale...” | Health-oriented creativity | Sustainable self-care practices | Empowerment and regaining agency |
| Study 4: Valachiné et al. (2022) | Online self-help art therapy-based tasks during COVID-19: Qualitative study | | |
| **Participant Quote or Description (from the study)** | **Initial Code** | **Descriptive Theme** | **Final Analytical Theme** |
| “It feels a bit frustrating, because I’ve been between these walls for a long time.” | Frustration from confinement | Emotional strain due to isolation | Emotional processing and expression through symbolic creation |
| “It is sad and devastating at the same time to imagine how we could have spent our spring break…” | Loss of opportunity | Mourning disrupted routines | Emotional processing and expression through symbolic creation |
| “A mix of being unsettled, while calm, but feeling like you might burst…” | Emotional turbulence | Conflicted internal states | Emotional processing and expression through symbolic creation |
| “The coronavirus situation delayed me with my studies…” | Academic disruption | Feeling out of control | Empowerment and regaining agency |
| “The current circumstances... have a big influence—which leads to me being ‘stuck’ in my childhood home.” | Lack of autonomy | Disrupted personal agency | Empowerment and regaining agency |
| “It feels hopeful. It feels like summer and warmth. I allowed myself to accept the memory and to let go a little.” (Colour task) | Acceptance and hope | Cognitive reappraisal | Transformation of trauma into post-traumatic growth |
| “It makes me calm and remember the sea sounds that I totally love…” (Squiggle task) | Nature-based calmness | Sensory grounding | Communal support and collective meaning-making |
| “Raised beautiful memories of my grandma. It raises tranquility in me more than anything.” (Object task) | Familial memory as emotional anchor | Emotional support through attachment | Communal support and collective meaning-making |
| “Safety to me is someone… always there holding your hands and making you feel safe.” (Safety task) | Interpersonal security | Imagined safe relationships | Adaptive communication and nonverbal connection |
| “The task gave me an opportunity to turn my frustrations into something more manageable.” (Transformation task) | Emotional regulation through creativity | Emotional catharsis | Transformation of trauma into post-traumatic growth |
| “It feels good to look at the new picture… feelings of control, power and self-confidence.” (Avatar task) | Empowerment through visual self-representation | Restoring control | Empowerment and regaining agency |
| “The dragon… rage and love… frustration of the lockdown, but longing for her love partner…” (Blot task) | Dual emotional states | Emotional ambivalence | Emotional processing and expression through symbolic creation |
| Study 5: Renzi et al. (2021) | Using drawings to express and represent one's emotional experience during the coronavirus disease 2019 pandemic: a case report of a woman living in a nursing home. | | |
| **Participant Quote or Description (from the study)** | **Initial Code** | **Descriptive Theme** | **Final Analytical Theme** |
| “She decided to give a name only to this drawing, titling it Beyond the hedge. This first drawing seems to represent her depressive feelings… Also seems to express hope—hope to be soon outside the room, beyond the hedge…” | Naming artwork with symbolic meaning; hope despite isolation | Hopeful symbolic expression of confinement and aspiration | Emotional processing and expression through symbolic creation |
| “The patient represented exactly what she saw when lying on her bed: the white wall, the window, and the view through the window—the hedge... and the vegetation beyond.” | Literal depiction of surroundings | Externalising isolation through familiar visual anchors | Emotional processing and expression through symbolic creation |
| “Drawing 2... a seascape… with a big grey skyscraper… continuing to limit her life.” | Visual metaphor of partial recovery and limitation | Representation of emotional transition using obstacles | Emotional processing and expression through symbolic creation |
| “Drawing 3… a seascape… shows a better emotional state… expresses positive emotions of serenity and peace.” | Imagery of calmness and openness | Representation of improved mood through peaceful imagery | Emotional processing and expression through symbolic creation |
| “During psychological counselling sessions, the patient discussed these drawings, and they were used as stimuli for exploring her emotional condition…” | Art used as a conversational prompt in therapy | Facilitating dialogue through artistic output | Adaptive communication and nonverbal connection |
| “Her mood slowly began to worsen… she had difficulties exploring and expressing her feelings verbally.” | Decline in mood; verbal communication challenges | Communication challenges during isolation | Adaptive communication and nonverbal connection |
| “Drawing... sustained her ability to explore, represent, express, and regulate her emotional condition.” | Emotional regulation via drawing | Emotional regulation using art | Transformation of trauma into post-traumatic growth |
| Study 6: Houari & Hadjoui (2022) | Disabled and Confined: Using Art Therapy as a Coping Strategy During the COVID-19 Pandemic | | |
| **Participant Quote or Description (from the study)** | **Initial Code** | **Descriptive Theme** | **Final Analytical Theme** |
| “Farah picked up the colouring pencils first. She drew discontinuous lines and put successive points on the paper.” | Limited engagement with materials; tentative exploration | Early emotional expression through art | Emotional processing and expression through symbolic creation |
| “Farah’s capacity and willingness to use clay to make objects was remarkable... she squeezed, rolled and formed shapes.” | Kinesthetic engagement; tactile connection | Use of clay as preferred medium for self-expression | Emotional processing and expression through symbolic creation |
| “Farah selected a song associated with childhood memories... appeared relaxed and at ease in the music session.” | Connection to memory; music as emotional regulator | Using music to evoke positive emotions and self-soothing | Reconnecting with identity and memory |
| “Farah...successfully imitated my body movements... this configuration of behaviour is clear in the feelings of satisfaction she had after accomplishing the task.” | Mimicry as expression; playful movement | Role-play promoting body awareness and emotional expression | Empowerment and regaining agency |
| “The act of holding pens and clay enhanced the movement of her hands... Through painting, Farah made physical exercise, released stress and spent enjoyable moments.” | Creative activity improving psychomotor skills | Art facilitating physical and emotional wellbeing | Empowerment and regaining agency |
| “Farah was invited to narrate a popular story... she was noticed to make efforts to pronounce words correctly; even the new vocabulary items.” | Narrative as self-expression; speech improvement | Storytelling supporting communication development | Adaptive communication and nonverbal connection |
| “The enjoyment she felt during the music session... stabilised mood was noticed after the session.” | Music stabilising mood | Music as emotional regulation | Emotional processing and expression through symbolic creation |
| “The happy ending of a family reunion amplified hope and reinforced the belief in a better future to come ahead.” | Symbolic hope through narrative | Storytelling as hope-building mechanism | Transformation of trauma into post-traumatic growth |
| “Farah’s parents reported that she had no violent reactions during the period of intervention... she was significantly less irritable at home.” | Reduction in agitation; improved behaviour | Art as a calming intervention during confinement | Transformation of trauma into post-traumatic growth |
| “Art therapy can provide a space for free dialogue and expression for people with disabilities... artistic expression can develop resilience and create the atmosphere for positive social change.” | Expression; inclusion; resilience | Art creating inclusive space and encouraging advocacy | Communal support and collective meaning-making |
| Study 7: Usiskin, M., & Lloyd, B. (2020). | Lifeline, frontline, online: adapting art therapy for social engagement across borders | | |
| **Participant Quote or Description (from the study)** | **Initial Code** | **Descriptive Theme** | **Final Analytical Theme** |
| “People became connected to each other through the use of objects and photos… carefully prepared… personal and communicative.” (Kate France, personal communication) | Connecting through shared symbols and images | Use of nonverbal cues to maintain relational bonds | Adaptive communication and nonverbal connection |
| “It reminded A of his childhood. A showed a picture of himself as a child… someone else added another connection… a chain thing.” | Recollection of memory through art | Reconnecting with memory and identity in collective settings | Emotional processing and expression through symbolic creation |
| “I think all these games are great, they are a gateway for being together… suddenly there is some emotion crossing the screen.” | Structured creative activities as gateways to connection | Playful, embodied art as a bridge for emotion and interaction | Adaptive communication and nonverbal connection |
| “All these different people in different places… communicating on a horizontal level.” | Shared art space breaking social hierarchies | Community-level participation across borders | Communal support and collective meaning-making |
| “It’s a virtual table… an in-between space which brings these possibilities with it.” | The online art space as a liminal social setting | Art as co-creation across borders during isolation | Communal support and collective meaning-making |
| “Coronaquilt… a patchwork narrative that seeks to help cope with personal feelings of anxiety, while building empathy for others’ experiences.” (Zucker, 2020 citing Art Refuge) | Collective creative ritual to cope with anxiety | Symbolic acts of empathy and solidarity | Transformation of trauma into post-traumatic growth |
| “Some participants reported that the Coronaquilt was helping them feel less alone in their situation.” | Collective expression reducing isolation | Shared creative processes to mitigate loneliness | Communal support and collective meaning-making |
| “Physical act of sewing… helping them feel more grounded, providing distraction and absorption in the here and now.” | Repetitive creative act for emotional regulation | Art as grounding and structuring time during crisis | Empowerment and regaining agency |
| “Online models… create viable spaces for social engagement… emotional resilience.” | Art spaces fostering resilience | Sustained psychosocial support via remote participation | Empowerment and regaining agency |
| “The original aim… was to keep communication lines open… What initially were intended as holding spaces have become viable creative spaces.” | Transition from containment to creativity | Development of agency and emotional articulation | Transformation of trauma into post-traumatic growth |
