# Supplementary figures and images for: Healing through art: a thematic synthesis within a quasi-systematic review of art’s impact on adult mental well-being during the COVID-19 pandemic
Source: BMC Public Health. 2025 May 3;25:1641. doi: 10.1186/s12889-025-22741-0 (PMC12048940; doi:10.1186/s12889-025-22741-0)

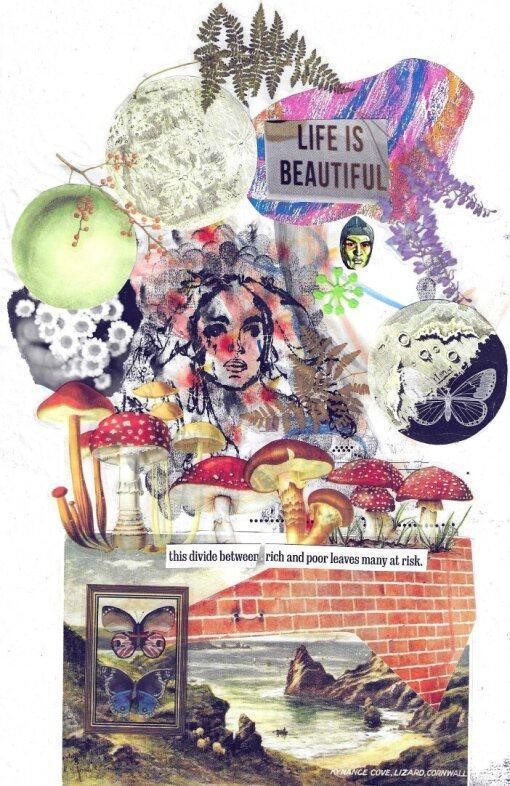

Supplement: Supplementary file 3 — Supplementary Material 3 [file 12889_2025_22741_MOESM3_ESM.jpg]
